# Nutrition Literacy, Hydration Awareness and Food Label Use in School Populations: A Cross‐Sectional Study of Students and Teachers Across European Contexts

**DOI:** 10.1111/jhn.70258

**Published:** 2026-04-30

**Authors:** Donatella Coradduzza, Giannagelo Boccuzzi, Giancarlo Masi, Christian Porstner, Raphaël Dubé, Nikolas Nikolaou, Stefania Sedda, Maja Merdovic, Serenella Medici, Ciriaco Carru, Roberto Solinas, Maria Grazia Pirina

**Affiliations:** ^1^ Department of Biomedical Sciences University of Sassari Sassari Italy; ^2^ Mine Vaganti NGO Sassari Italy; ^3^ NGO NEST Berlin e.V. Berlin Germany; ^4^ Université des Antilles Guadeloupe France; ^5^ Department of Education University of Cyprus Nicosia Cyprus; ^6^ Institute for Public Health of Serbia Belgrade Serbia; ^7^ Department of Chemical, Physical, Mathematical and Natural Sciences University of Sassari Sassari Italy

**Keywords:** food labelling, health promotion, nutrition literacy, hydration, nutrition literacy, public health nutrition, school health, sustainability

## Abstract

**Background:**

Nutrition literacy is increasingly recognised as a key determinant of dietary behaviours and long‐term public health outcomes. Schools represent strategic settings for strengthening nutrition literacy‐related competencies, including hydration awareness and food label use, yet comparative. However, descriptive cross‐country evidence across European school contexts remains limited.

**Methods:**

A cross‐sectional, school‐based survey was conducted among 432 students (aged 11–16 years) and 108 teachers across five European contexts (Italy, Germany, Cyprus, Serbia and Guadeloupe [France]). Culturally adapted questionnaires developed within the Erasmus+ GARDENS project assessed predefined domains of nutrition knowledge (food groups, macronutrient functions, fibre, vitamins and minerals), hydration awareness, food label use and selected sustainability‐related concepts. Knowledge items were coded dichotomously (correct/incorrect), and behavioural indicators were analysed descriptively. Cross‐country comparisons were exploratory.

**Results:**

Teachers in Germany and Italy reported higher levels of hydration awareness (> 90%) and food label use (≥ 75%), whereas lower self‐reported food label literacy was observed in Guadeloupe (48%). Among students, incorrect responses to nutrient‐function items (defined as misconceptions) were observed in 40% of the overall sample, with higher proportions in Guadeloupe and Serbia. Cross‐country variability was present across all assessed domains.

**Conclusions:**

Marked substantial descriptive differences in nutrition literacy knowledge, hydration awareness and food label use were observed across school populations in selected European contexts. These findings provide baseline public health evidence supporting the integration of nutrition literacy—particularly hydration and food labelling—into school‐based health promotion strategies, with attention to equity and contextual factors. These findings provide baseline evidence to inform school‐based health promotion strategies and highlight the relevance of contextual and equity‐related considerations. Further research using validated instruments and inferential designs is warranted.

## Summary

- Higher levels of teachers' nutrition literacy co‐occur with greater student awareness, hydration awareness and food label understanding.
- Cross‐country disparities highlight the need for equity‐oriented school nutrition strategies.
- Integrating food labelling and hydration education into school routines may strengthen public health prevention.

## Graphical abstract

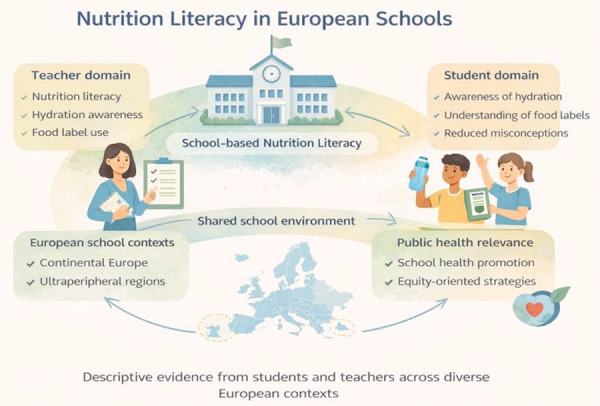

## 1 Introduction

Improving nutritional health and promoting environmentally sustainable food behaviours among children and adolescents are recognised priorities in public health nutrition and preventive health strategies worldwide [1, 2, 3]. Across Europe, unhealthy dietary patterns and suboptimal adherence to evidence‐based dietary recommendations contribute substantially to the burden of non‐communicable diseases, while unsustainable food consumption practices further exacerbate environmental and social challenges [4, 5, 6]. Schools are increasingly viewed as strategic settings for public health action because they represent daily food environments and learning contexts in which nutrition‐related knowledge and routine practices can be shaped during critical developmental stages [7].

Recent policy initiatives, including the European Commission's Farm to Fork Strategy within the Green Deal framework, highlight education as a lever to support healthier and more sustainable food systems [8, 9, 10, 11, 12]. However, translating these policy ambitions into effective school‐based practice remains challenging [13]. Evidence indicates persistent gaps in food‐related knowledge among school‐aged populations, including misunderstandings about macronutrient functions, limited awareness of food processing and inconsistent attention to hydration and physical activity [14, 15, 16, 17]. At the same time, sustainability‐related aspects of food—such as food labelling, environmental impact and responsible consumption—are variably addressed within school initiatives [18].

Nutrition literacy, broadly defined as the capacity to access, understand, appraise and apply nutrition‐related information in ways that support informed dietary decisions, has emerged as a key public health construct underpinning health‐related behaviours and long‐term outcomes [19, 20, 21, 22]. Within school settings, this construct is particularly relevant, as it reflects competencies that can be developed through structured education and reinforced through daily routines [23].

Although the constructs of nutrition literacy and food literacy overlap conceptually, the present study focused specifically on nutrition literacy because the primary domains assessed (e.g., macronutrient knowledge, hydration awareness and interpretation of nutrition labels) relate directly to understanding and applying nutrition‐related information rather than broader food system competencies such as food preparation, procurement or socio‐cultural dimensions of eating [24]. This conceptual focus allowed for clearer operationalisation within a school‐based survey context and facilitated consistent cross‐country comparison of health‐oriented competencies [25].

The present study was conducted within the framework of the Erasmus+ GARDENS project (Greening Actions for Resilient, Diverse and Empowered Nutrition in Schools), a European multinational initiative aimed at strengthening nutrition literacy and sustainable food practices in school communities across diverse national contexts.

A core rationale for the present work is the need for cross‐country descriptive evidence that characterises nutrition literacy–related domains among students and teachers while accounting for contextual diversity across European school settings [26]. Comparative assessment is particularly relevant in Europe, where education systems, nutrition education policies, school health promotion practices and food labelling environments vary across countries and regions [27]. Despite shared European‐level policy directions, empirical evidence directly comparing school‐based indicators of nutrition literacy, hydration awareness and food label use across diverse contexts remains limited [28]. Cross‐country descriptive data may help identify converging strengths and context‐specific gaps, thereby informing equity‐oriented educational strategies and supporting the design of future interventions that are adaptable to local school systems [29].

The inclusion of hydration awareness and food label use was motivated by their relevance as actionable, routine‐linked components of nutrition literacy in school life [30]. Hydration behaviours are shaped by everyday school conditions—such as access to water fountains, permission to keep water bottles in class, scheduled breaks and sports participation—making hydration education a practical target for health‐promoting routines [31]. Similarly, food labels represent a frequent point of contact with nutrition information for families and adolescents; label engagement can be directly linked to school‐relevant situations such as interpreting packaged snacks, comparing beverages or evaluating school canteen/packed‐lunch options [32]. Strengthening these applied competencies can support healthier decision‐making and align with prevention‐oriented approaches in school settings [33].

To address these aims, culturally adapted questionnaires were used to assess key nutrition literacy–related domains, including knowledge of food groups and macronutrients, hydration awareness, food label use and familiarity with selected sustainability concepts relevant to everyday food choices [34, 35, 36]. Misconceptions in nutrition and sustainability often arise from fragmented information and informal learning processes, and may become entrenched over time, limiting the effectiveness of later educational interventions [37]. Moreover, the integration of nutrition and sustainability education into school curricula remains uneven across countries, frequently confined to non‐compulsory modules or short‐term initiatives rather than embedded, longitudinal approaches [38, 39, 40, 41, 42].

By presenting a comparative analysis of data collected from students and teachers across multiple European contexts, this study aims to identify converging patterns and critical gaps in school‐based nutrition literacy. The findings are intended to support the development of equity‐oriented, school‐based strategies in public health nutrition, with particular attention to hydration and food labelling as practical and actionable components of nutrition literacy within everyday school environments.

**Figure 1:**
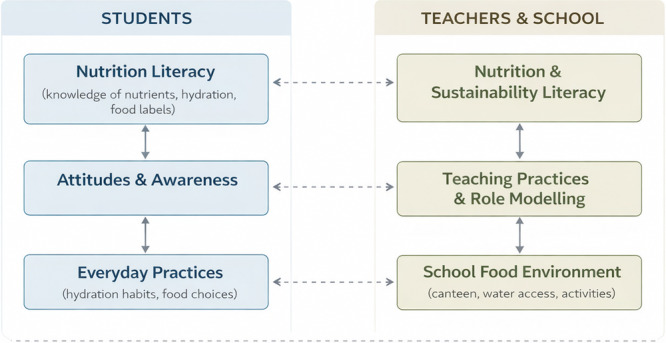
Conceptual framework illustrating the reciprocal relationships between student and teacher domains in school‐based nutrition and sustainability contexts.

The conceptual framework guiding the selection and interpretation of the assessed domains is presented in Figure 1.

## 2 Methods

### 2.1 Study Design and Context

This study employed an exploratory, cross‐sectional, multicountry school‐based survey design conducted within the framework of the Erasmus+ GARDENS project (Grant Agreement No. 2023‐1‐FR01‐KA220‐SCH‐000160068), a European Union co‐funded initiative aimed at promoting eco‐healthy nutritional attitudes and sustainability awareness among school populations. The project was conceptually informed by the European Commission's Farm to Fork Strategy and by the World Health Organization (WHO) Health Promoting Schools framework, which emphasises integrated, whole‐school approaches to health education and supportive learning environments [43, 44, 45]. Data were collected in five contexts—Italy, Germany, Cyprus, Serbia and Guadeloupe (France)—with the objective of describing nutrition literacy–related domains among students and teachers across heterogeneous European school settings. The selection of participating contexts followed a purposive heterogeneity rationale designed to capture variation in: (i) geographic regions (Southern, Central and Eastern Europe, alongside an EU ultraperipheral region); (ii) educational systems and school health implementation frameworks; and (iii) dietary traditions and food environments. This included Mediterranean‐influenced settings (Italy and Cyprus) and non‐Mediterranean contexts (Germany and Serbia). Guadeloupe, an overseas department of France and an ultraperipheral region of the European Union, was included to reflect a structurally distinct food environment characterised by geographic remoteness and documented reliance on imported foods—features commonly described for EU outermost regions. Such characteristics may plausibly influence exposure to food labelling, availability of processed versus fresh foods, and sustainability‐related purchasing behaviours in everyday life. Given the descriptive nature of the survey and its focus on school‐based literacy domains, structural factors specific to Guadeloupe (e.g., import dependency or food price variability) were not assessed through direct economic indicators. Instead, they were explored indirectly through proxy questionnaire items capturing school‐ and household‐relevant behaviours and applied competencies potentially sensitive to contextual food environments. These included items assessing routine food label use (e.g., “Do you make a habit of reading food labels on food packages?”), recognition of food processing categories (e.g., “Do you know the difference between whole and processed foods?”), sustainability‐related purchasing practices (e.g., frequency of organic product purchases) and identification of sugar‐containing beverages or packaged snack consumption at breakfast. The study was designed to characterise baseline descriptive patterns across contexts rather than to establish causal relationships or evaluate intervention effects.

### 2.2 Survey Development and Operational Definitions

Two structured, age‐appropriate questionnaires were developed: one for students aged 11–16 years and one for primary and lower secondary school teachers [46]. Both instruments—hereafter referred to consistently as “questionnaires”—were original tools developed within the Erasmus+ GARDENS project by a multidisciplinary panel of experts from the University of Sassari and project partner institutions, including specialists in clinical nutrition, public health, biochemistry, sustainability sciences and education.

#### 2.2.1 Development Process

The development process followed four structured phases, informed by current literature on nutrition literacy, hydration awareness and sustainable dietary patterns [47, 48].

##### 2.2.1.1 Phase 1—Domain Identification

Core domains were identified based on the literature on nutrition literacy, health literacy, hydration education and sustainable dietary practices.

##### 2.2.1.2 Phase 2—Item Drafting and Adaptation

Items were adapted from previously validated nutrition knowledge and health behaviour questionnaires (e.g., Mediterranean diet adherence tools, nutrition knowledge surveys) and reformulated for school‐level comprehension. Where no suitable validated items were available (e.g., sustainability‐related knowledge in school settings), new items were developed.

##### 2.2.1.3 Phase 3—Expert Consultation Rounds

Two iterative rounds of structured review were conducted among project experts to assess content relevance, clarity, age appropriateness and cross‐country comparability. Revisions were made until consensus was reached. Content validity was established through expert consensus.

##### 2.2.1.4 Phase 4—Pilot Testing and Linguistic Validation

The questionnaires were pilot‐tested in each participating country (*n* ≈ 15–25 per site) to assess comprehension and feasibility. Translation followed forward–backward procedures to ensure semantic and conceptual equivalence. All questionnaires were translated into local languages and reviewed to ensure clarity, cultural appropriateness and comprehension by the target populations [49, 50, 51, 52, 53, 54, 55, 56, 57].

The teacher questionnaire addressed analogous domains with additional higher‐level nutrition science items, including energy density, fibre typology, obesity classification, macronutrient functions and sustainability‐related concepts [58, 59, 60, 61, 62].

The student instrument assessed foundational knowledge and awareness domains appropriate for ages 11–16, whereas the teacher instrument included items reflecting expected greater disciplinary knowledge. Hydration awareness was included within the health behaviour domain in both instruments.

Self‐reported weight and height were collected and body mass index (BMI) was calculated (kg/m^2^). BMI was categorised according to age‐ and sex‐specific reference standards [63, 64].

Formal psychometric testing (e.g., exploratory or confirmatory factor analysis and internal consistency indices such as Cronbach's *α*) was not performed in this baseline cross‐country implementation and will be addressed in future validation studies [65, 66].

The full item wording, response formats and scoring structure are provided in Supporting Information (Online Resources 2 and 3) to ensure transparency and reproducibility.

###### 2.2.1.4.1 Operational Definitions

For the purposes of this study, the following operational definitions were adopted:

*Nutrition literacy* was conceptualised as the ability to access, understand, appraise and apply nutrition‐related information in ways that support informed dietary decisions [20, 21, 67, 68].
In this study, it operationally encompassed knowledge of food groups, macronutrients, fibre, vitamins, minerals, hydration awareness and interpretation of nutrition‐related information relevant to everyday food choices.
*Food label literacy* was defined as self‐reported familiarity with, and use of, food labels to identify nutritional information (e.g., energy content, key nutrients) and to distinguish between whole, minimally processed and processed foods. Given cross‐country variation in front‐of‐pack labelling systems (e.g., Nutri‐Score adoption differences), items were phrased generically (e.g., “Do you read food labels?”) and did not reference specific national labelling formats. Question wording remained conceptually identical across countries. Responses were treated descriptively: self‐reported label use was analysed as frequency percentages; no objective assessment of label interpretation accuracy was performed; and no competency score was calculated.
*Hydration awareness* referred to awareness of the importance of adequate daily water intake as part of a healthy lifestyle, informed by WHO and EFSA guidance on adequate fluid intake for children and adolescents [49, 50]. Items assessed recognition of the importance of water, daily water intake categories and identification of high‐sugar beverages. This construct captured recognition of hydration needs rather than objective measures of fluid intake, and reported intake was not evaluated against national adequacy thresholds.
*Misconceptions about nutrients* were defined as incorrect responses to predefined knowledge items (e.g., attributing energy provision to vitamins instead of carbohydrates). Examples of such items are provided in Supporting Information [51].
*Sustainability‐related knowledge* included awareness of food‐related environmental concepts—specifically, sustainable diet definition, responsible consumption, eco‐conscious purchasing and food waste awareness—informed by sustainability education literature and European school‐based health frameworks [55, 56, 57]. Behavioural implementation was not assumed.

### 2.3 Participants and Recruitment

Participants were recruited from primary and lower secondary schools participating in the project between November 2023 and February 2025. Schools were identified through collaboration with project partner institutions in each participating country. Within the eligible pool of schools, selection was performed using a structured random allocation procedure to minimise selection bias. Participation remained voluntary at the institutional level.

The final sample included 432 students (aged 11–16 years) and 108 teachers across the five contexts (Table 1).

**Table 1:** Participant characteristics by country.

Characteristic	Italy	Germany	Cyprus	Serbia	Guadeloupe	Total
Students, *n*	95	88	82	91	76	432
Age range (years)	11–16	11–16	11–16	11–16	11–16	11–16
Teachers, *n*	24	22	20	22	20	108
School level	Primary/Lower secondary	Primary/Lower secondary	Primary/Lower secondary	Primary/Lower secondary	Primary/Lower secondary	—
BMI data collected	Yes	Yes	Yes	Yes	Yes	Yes
Data collection period	Nov 2023–Feb 2025	Nov 2023–Feb 2025	Nov 2023–Feb 2025	Nov 2023–Feb 2025	Nov 2023–Feb 2025	—

*Note:* Sample sizes per country are indicative. BMI was calculated from self‐reported weight and height and categorised per age‐ and sex‐specific reference standards. Missing data handled at item level (available‐case analysis).

Although random selection was applied within the eligible pool, the sample cannot be considered fully nationally representative, as participation was voluntary and no stratified national sampling framework was employed.

Eligibility criteria required enrolment as a student or current teaching role within a participating school. Written informed consent was obtained from all teachers. For students, parental consent and pupil assent were secured in accordance with national and institutional regulations in each country.

Participants were recruited from primary and lower secondary schools participating in the Erasmus+ GARDENS project between November 2023 and February 2025.

Schools were approached through project partner networks in each country. Within collaborating institutions, a structured allocation procedure was applied; however, participation remained voluntary at the institutional level. The sampling approach, therefore, corresponds to a pragmatic multicentre convenience sample.

#### 2.3.1 Eligibility Criteria

- Students:

✓ Aged 11–16 years
✓ Enroled in participating schools

- Teachers:

✓ Currently employed in participating schools
✓ Involved in classroom teaching

Eligibility was verified by school administrative records.

##### 2.3.1.1 Ethical Considerations

The study involved anonymous, non‐interventional questionnaire‐based data collection within educational settings and did not include clinical procedures, biological sampling or sensitive health data. According to national regulations in participating countries for school‐based educational surveys, formal ethics committee approval was not required.

Participation was voluntary at all levels. Written informed consent was obtained from all teachers. For students, written parental consent and student assent were secured in accordance with national and institutional regulations in each country.

Data were collected anonymously and processed in compliance with the General Data Protection Regulation (GDPR, EU 2016/679). No identifiable personal data were retained.

### 2.4 Data Collection and Analysis

Surveys were administered either in online or paper‐based format, depending on local school infrastructure and digital accessibility. To ensure cross‐country consistency and methodological rigour, standardised procedures were implemented across all participating sites. These included uniform written administration instructions provided to local coordinators, the use of standardised response coding manuals and harmonised data entry templates. Data were centrally collated and underwent double‐check verification to minimise transcription errors. Predefined coding criteria were applied consistently across countries prior to analysis.

Analyses were not stratified by educational level (primary vs. lower secondary), as the exploratory objective of the study focused on cross‐country patterns within the overall school sample. Given the sample size within individual countries, stratified analyses may have reduced interpretability and statistical stability.

The study was conducted and reported in accordance with the STROBE guidelines for cross‐sectional studies. The completed STROBE checklist is provided as Data S1 [69].

Knowledge‐based items were coded dichotomously (1 = correct; 0 = incorrect). For items allowing multiple correct responses, predefined scoring rules were applied, as detailed in the Supporting Information. Behavioural and perception‐based items were analysed descriptively without normative scoring.

Frequencies and proportions were calculated by country and participant group (students and teachers). Given the exploratory nature of the study, no inferential statistical testing or causal modelling was performed.

All analyses were conducted using SPSS (Version 27). Missing data were handled using available‐case analysis at the item level without imputation. The study is reported in accordance with the STROBE guidelines for cross‐sectional studies, and the completed checklist is provided as Supplementary Material S1 – STROBE Checklist for Cross‐sectional Studies.

## 3 Results

### 3.1 Teacher Knowledge of Nutrition, Hydration and Sustainability

A total of 432 students (aged 11–16 years) and 108 teachers participated across five countries (Italy, Germany, Cyprus, Serbia and Guadeloupe [France]). The distribution of student and teacher participants across countries is shown in Table 2. Teachers across the five participating countries demonstrated generally high awareness of core nutrition and health principles. Hydration awareness was particularly strong in Germany (94%) and Italy (92%). Food label literacy was also relatively high in Germany (80%) and Italy (75%). In contrast, teachers in Guadeloupe reported substantially lower levels of food label literacy (48%). Sustainability awareness was reported at comparable levels across most contexts, with Germany and Italy among the highest‐performing countries.

**Table 2:** Distribution of student and teacher participants by country (*n* = 540 total respondents).

Country	Students (*n*)	Teachers (*n*)
Germany	90	22
Italy	95	25
Cyprus	80	20
Serbia	85	21
Guadeloupe	82	20
Total	432	108

### 3.2 Student Awareness and Eating Behaviours

Student knowledge of nutrition concepts varied considerably across countries. Misconceptions about nutrient functions were most prevalent in Guadeloupe (58%) and Serbia (47%), compared with lower levels in Germany (28%) and Cyprus (32%). Student hydration awareness reflected that of teachers, with Italy and Germany again achieving the highest levels.

### 3.3 Teacher‐Reported Indicators

Teacher hydration awareness was calculated as the proportion of respondents correctly identifying recommended daily fluid intake ranges and reporting awareness of hydration importance.

Teacher food label literacy was calculated as the proportion of respondents reporting familiarity with and routine use of nutrition labels.

Across countries:

- Germany: Hydration awareness 94%; food label literacy 80%.
- Italy: Hydration awareness 92%; food label literacy 75%.
- Guadeloupe: Food label literacy 48%.

### 3.4 Cross‐Country Comparison

When comparing responses across all five countries, Germany consistently reported the highest combined scores on both teacher and student indicators of nutrition and sustainability literacy. Italy followed closely, particularly in hydration awareness and food label literacy. Guadeloupe and Serbia reported the most pronounced gaps, characterised by higher proportions of student misconceptions and lower teacher food label literacy. Sustainability awareness was broadly similar across contexts.

Across all assessed indicators:

- Germany reported the highest combined percentages for teacher hydration awareness (94%), teacher food label literacy (80%) and lower student misconception rates (28%).
- Italy reported high hydration awareness (92%) and food label literacy (75%).
- Guadeloupe reported the highest student misconception rate (58%) and the lowest teacher food label literacy (48%).

No inferential comparisons were conducted.

### 3.5 Graphical Results

**Figure 2:**
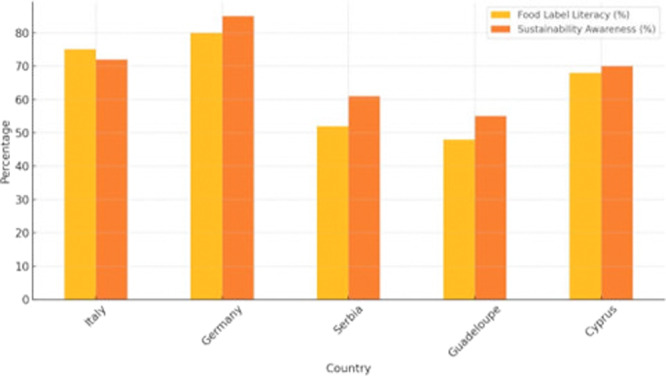
Teacher‐reported food label literacy (%) and sustainability awareness (%) by country (*n* = 108 teachers). Percentages calculated using country‐specific teacher denominators.

Figure 2 presents the comparative percentages of food label literacy and sustainability awareness among teachers across the five surveyed countries. Germany and Italy reported the highest values across both domains. Lower percentages were observed in Guadeloupe and Serbia.

**Figure 3:**
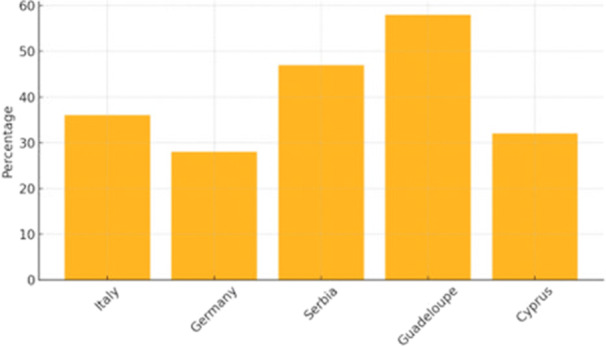
Prevalence of student misconceptions about nutrient functions (%) by country (*n* = 432 students). Misconceptions defined as incorrect responses to predefined nutrient‐function items.

Figure 3 shows the prevalence of misconceptions about nutrient functions among students across the five surveyed countries. The highest proportions of incorrect responses were observed in Guadeloupe (58%) and Serbia (47%), whereas Germany (28%) and Cyprus (32%) reported the lowest proportions.

**Figure 4:**
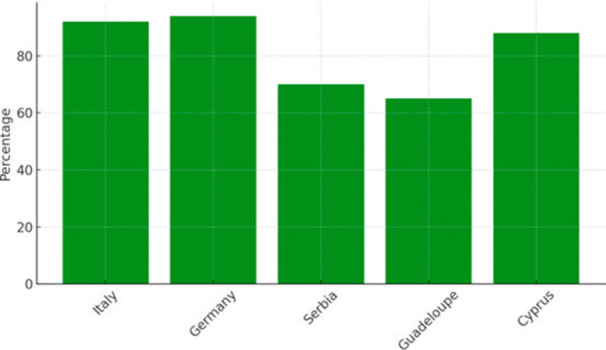
Student hydration awareness (%) by country (*n* = 432 students). Percentages reflect students correctly identifying recommended hydration practices.

Figure 4 illustrates student hydration awareness levels by country. Italy and Germany reported the highest student awareness scores. Lower awareness levels were observed in Guadeloupe and Serbia.

Italy and Germany reported the highest hydration awareness levels among both teachers and students. Guadeloupe and Serbia reported lower awareness levels in both groups. No inferential testing was conducted; the parallel patterns observed across groups are descriptive only.

### 3.6 Statistical Summary of Survey Metrics

Descriptive statistics for the main indicators assessed in the Erasmus+ GARDENS project are presented in Table 3. The analysis summarises measures of central tendency and dispersion for food label literacy, sustainability awareness, misconceptions about nutrients and hydration awareness across the five participating countries. All values are expressed as percentages.

**Table 3:** Descriptive statistics (mean, standard deviation and quartiles) across five participating countries for key survey dimensions: Food label literacy, sustainability awareness, misconceptions about nutrients and hydration awareness.

Metric	Number of countries (*n* = 5)	Mean (%)	SD	Min	25%	50%	75%	Max
Food label literacy (%)	5	64.6	14.06	48.0	52.0	68.0	75.0	80.0
Sustainability awareness (%)	5	68.6	11.46	55.0	61.0	70.0	72.0	85.0
Misconceptions about nutrients (%)	5	40.2	12.21	28.0	32.0	36.0	47.0	58.0
Hydration awareness (%)	5	81.8	13.35	65.0	70.0	88.0	92.0	94.0

Overall, Germany and Italy consistently reported the strongest outcomes across assessed domains, particularly in hydration awareness and food label literacy. Guadeloupe and Serbia showed the most pronounced gaps, with higher student misconception rates and lower teacher food label literacy. Cyprus reported intermediate values: teacher hydration awareness 72%, student misconception rate 32%, placing it between higher‐performing contexts (Germany: 94%, 28%; Italy: 92%, 30%) and lower‐performing contexts (Serbia: 65%, 47%; Guadeloupe: 88%, 58%) (Table 4).

**Table 4:** Summary of key findings on teacher and student nutrition and sustainability literacy across countries.

Country	Teacher hydration awareness (%)	Teacher food label literacy (%)	Student misconceptions about nutrients (%)	Student hydration awareness (%)
Germany	94	80	28	High
Italy	92	75	N/A (lower range, ~30 s)	High
Cyprus	Moderate (~70 s)	Moderate (~ 60 s)	32	Moderate–High
Serbia	Lower (~ 60 s)	Lower (~ 55–60)	47	Lower–Moderate
Guadeloupe (FR)	88 (teachers), lower for students	48	58	Lower

**Country**	**Teacher hydration awareness (%)**	**Teacher food label literacy (%)**	**Student misconceptions (%)**	**Student hydration awareness (%)**
Germany	94	80	28	90
Italy	92	75	30	88
Cyprus	72	60	32	74
Serbia	65	57	47	68
Guadeloupe	88	48	58	70

## 4 Discussion

The observed cross‐country variability should be interpreted within the broader educational, cultural and socio‐economic contexts of the participating countries. Although the present study was not designed to establish causal explanations, differences in nutrition literacy–related domains may plausibly reflect variations in national nutrition education policies, the extent to which structured nutrition and sustainability content is embedded within school curricula and differences in teacher training frameworks. Cultural dietary traditions, such as stronger adherence to Mediterranean dietary models in Southern European contexts, may also contribute to baseline familiarity with core nutrition concepts. Conversely, structural constraints—including geographic remoteness, food system dependency on imports or socio‐economic disparities—may influence exposure to healthy foods and sustainability‐related information. Media discourse and public policy emphasis on environmental sustainability and healthy eating may further shape awareness among both students and teachers. These interpretations remain hypothesis‐generating and descriptive, consistent with the exploratory nature of the study, and should be examined in future research using stratified sampling designs and inferential modelling approaches.

This cross‐sectional study provides comparative evidence on nutrition literacy, hydration awareness and food label use among students and teachers across selected European school contexts. The knowledge domains assessed included understanding of food groups, macronutrient functions, fibre and micronutrients, recognition of processed versus whole foods, hydration awareness and self‐reported food label use. Differences were observed across these specific domains, highlighting substantial between‐country variability with coexisting strengths and gaps that are relevant for public health nutrition and school‐based health promotion.

In particular, variability was noted in (i) macronutrient‐function knowledge, (ii) prevalence of misconceptions about nutrient roles, (iii) hydration awareness and (iv) reported engagement with food labels. These findings suggest heterogeneous implementation and reinforcement of nutrition‐related competencies within European school environments.

From a public health perspective, these findings are relevant given the established contribution of unhealthy dietary patterns to the burden of non‐communicable diseases in childhood and later life [54, 70]. Schools represent daily food and learning environments in which nutrition literacy skills—such as understanding food labels and recognising the importance of adequate hydration—can be supported during critical developmental stages.

**Figure 5:**
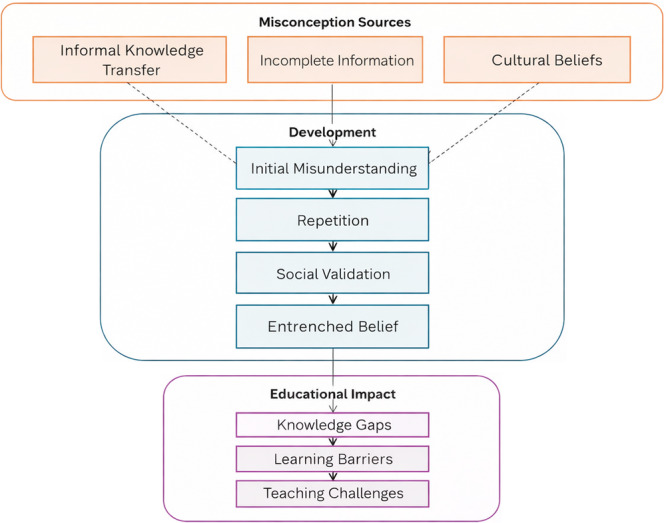
Conceptual framework illustrating potential pathways through which fragmented information exposure, inconsistent curricular integration and socio‐environmental factors may contribute to the formation of nutrition‐related misconceptions and influence their educational impact in school contexts. The framework is heuristic and does not represent tested causal pathways. Strengths and knowledge anchors.

A conceptual overview of the potential mechanisms underlying the formation of nutrition‐related misconceptions and their educational impact is presented in Figure 5. The figure is intended as a heuristic framework to illustrate how fragmented information exposure, inconsistent curricular integration, socio‐environmental influences and informal learning channels may interact in shaping student understanding. It does not represent tested causal pathways within this study but serves to contextualise the descriptive findings and guide future hypothesis‐driven research.

High levels of hydration awareness observed in several countries, particularly Italy, Germany and Cyprus, represent an encouraging finding and align with international recommendations for health promotion in youth. Adequate hydration has been associated not only with physical health but also with cognitive functioning and well‐being in school‐aged populations. Similarly, higher food label literacy reported by teachers in some contexts may provide a supportive foundation for integrating practical nutrition literacy skills into school routines.

Cultural dietary models, such as the Mediterranean diet in Italy and Cyprus, may contribute to these strengths by reinforcing nutrition‐related knowledge and attitudes within both school and community settings. Comparable initiatives, including farm‐to‐school and school garden programmes reported in other regions, have demonstrated potential benefits for nutrition knowledge and food awareness, supporting the relevance of experiential approaches within school‐based nutrition strategies [32].

### 4.1 Persistent Gaps and Equity Considerations

Despite these strengths, the study also identified persistent gaps, particularly misconceptions about basic nutrient functions and limited use of food labels among students in some contexts. Such gaps may constrain the ability of young people to navigate increasingly complex food environments and to make informed dietary choices.

These challenges appear more pronounced in socio‐economically disadvantaged or geographically remote settings, including ultraperipheral regions such as Guadeloupe, where reliance on imported foods and reduced access to fresh produce may limit opportunities for nutrition education and healthy food exposure. Similar patterns have been described in other low‐resource or rural school settings, highlighting the importance of equity‐oriented approaches in school‐based public health nutrition [33].

### 4.2 Schools as Public Health Platforms

Taken together, the findings reinforce the role of schools as important platforms for public health nutrition. Effective school‐based strategies require more than isolated educational activities; they depend on the alignment of curricula, daily school practices and the broader food environment. Teachers play a key role within this system, yet the reported variability in nutrition and sustainability literacy suggests that additional support and training may be needed to strengthen their capacity to address these topics consistently.

Embedding practical components—such as hydration routines, food label use and experiential learning activities—within existing school structures may help translate nutrition literacy concepts into everyday practice. Such approaches are consistent with the World Health Organization's Health Promoting Schools framework, which emphasises the integration of curriculum, school environment and with European policy priorities, while remaining adaptable to local contexts and resources community engagement in fostering sustainable health behaviours [71, 72].

### 4.3 Limitations

In addition, the lack of fully standardised instruments for assessing nutrition literacy and sustainability across Europe represents an ongoing methodological challenge. Although anthropometric data were collected and BMI calculated, analyses exploring associations between BMI and literacy domains were beyond the scope of the present manuscript. The inclusion of both primary and lower secondary school students introduces potential variability related to cognitive development and educational exposure. Analyses were not stratified by educational level, which may limit the ability to detect developmental differences in nutrition literacy domains. Nevertheless, the study provides valuable baseline data to inform future longitudinal and interventional research 36.

### 4.4 Implications for Public Health Nutrition

By identifying cross‐country patterns and gaps in nutrition literacy, hydration awareness and food label use, this study contributes to the evidence base supporting school‐based approaches to public health nutrition. The findings suggest that strengthening nutrition literacy—particularly through practical skills such as food labelling and hydration awareness—may represent a feasible and context‐sensitive strategy within school settings. Future research should build on this baseline evidence to evaluate targeted interventions and to explore how school environments, teacher training and broader structural factors interact to influence nutrition‐related outcomes among children and adolescents.

## 5 Conclusion

This study highlights variability in nutrition and sustainability‐related competencies across school populations in selected European contexts. While hydration awareness was generally high, gaps in nutrient knowledge and food labelling literacy remain evident, particularly in settings facing structural constraints. These findings support the relevance of nutrition literacy within school‐based public health strategies and indicate that schools may represent important environments for strengthening practical nutrition‐related competencies. Future research should build on this baseline evidence to inform equitable school‐based approaches aligned with international public health frameworks.

## Author Contributions

Conceptualization and project administration: Donatella Coradduzza and Giannangelo Boccuzzi. Methodology and formal analysis: Donatella Coradduzza and Giancarlo Masi. Investigation and data collection: Christian Porstner, Raphaël Dubé, Nikolas Nikolaou and Maja Merdovic. Writing – original draft: Donatella Coradduzza, Giannangelo Boccuzzi and Giancarlo Masi. Writing – review and editing: Donatella Coradduzza, Giannangelo Boccuzzi, Giancarlo Masi, Roberto Solinas and Maria Grazia Pirina. Supervision: Donatella Coradduzza and Serenella Medici. Resources and funding acquisition: Donatella Coradduzza, Roberto Solinas and Maria Grazia Pirina. Final approval of manuscript: All authors.

## Ethics Statement

Ethics approval: In accordance with national legislation and institutional requirements across all participating countries, no ethics committee review was required because this was an anonymous, minimal‐risk survey that did not involve clinical procedures and analyses were conducted on aggregated data. The study complied with the Declaration of Helsinki. Written informed consent was obtained from teachers; for students, parental consent and pupil assent were obtained prior to participation. Data handling complied with the EU General Data Protection Regulation (Regulation [EU] 2016/679) and applicable national laws. Authorisation to conduct the survey was granted by the relevant school authorities at each site.

## Conflicts of Interest

The authors declare no conflicts of interest.

## Supporting information

Supporting File:

## Data Availability

The data that support the findings of this study are available on request from the corresponding author. The data are not publicly available due to privacy or ethical restrictions. De‐identified dataset, codebook, Supplementary Material – STROBE Checklist for Cross‐sectional Studies and analysis scripts are available upon reasonable request from the corresponding author.
