# Supplementary material for: Nutrition Literacy, Hydration Awareness and Food Label Use in School Populations: A Cross‐Sectional Study of Students and Teachers Across European Contexts
Source: J Hum Nutr Diet. 2026 Apr 30;39:e70258. doi: 10.1111/jhn.70258 (PMC13133250; doi:10.1111/jhn.70258)
Supplement: Supplementary file 1 — Supporting File: [file JHN-39-0-s001.docx]

Supplementary Material – STROBE Checklist for Cross-sectional Studies

| **Item No.** | **Recommendation (STROBE)** | **Section of Manuscript** | **Page(s)** |
| --- | --- | --- | --- |
| 1a | Indicate the study’s design with a commonly used term in the title or the abstract | Title, Abstract | p. 1 |
| 1b | Provide in the abstract an informative and balanced summary of what was done and found | Abstract | p. 2 |
| 2 | Explain the scientific background and rationale | Introduction | pp. 3–4 |
| 3 | State specific objectives | Introduction | p. 4 |
| 4 | Present key elements of study design early | Methods – Study Design | p. 5 |
| 5 | Describe the setting, locations, and relevant dates | Methods – Study Design and Context | p. 5 |
| 6a | Give eligibility criteria and sources/methods of selection | Methods – Participants and Recruitment | p. 6 |
| 6b | For matched studies, give matching criteria and numbers | N/A | – |
| 7 | Define outcomes, exposures, predictors, confounders, effect modifiers | Methods – Survey Development | pp. 5–6 |
| 8 | For each variable, give data sources and measurement methods | Methods – Survey Development | pp. 5–6 |
| 9 | Describe efforts to address potential sources of bias | Limitations | p. 20 |
| 10 | Explain how the study size was arrived at | Methods – Participants and Recruitment | p. 6 |
| 11 | Explain handling of quantitative variables | Methods – Data Collection and Analysis | p. 7 |
| 12a | Describe all statistical methods, including those used to control for confounding | Methods – Data Collection and Analysis | p. 7 |
| 12b | Describe methods used to examine subgroups and interactions | N/A | – |
| 12c | Explain how missing data were addressed | Methods – Data Collection and Analysis | p. 7 |
| 12d | If applicable, describe analytical methods accounting for sampling strategy | Methods – Data Collection and Analysis | p. 7 |
| 12e | Describe any sensitivity analyses | N/A (exploratory study) | – |
| 13a | Report numbers of individuals at each stage | Results – Participants | p. 8 |
| 13b | Give reasons for non-participation at each stage | N/A | – |
| 13c | Consider use of a flow diagram | Supplementary Figure S1 (if available) | – |
| 14a | Give characteristics of study participants | Results | pp. 8–10 |
| 14b | Indicate number with missing data for each variable of interest | Results – Statistical Summary | p. 12 |
| 15 | Report numbers of outcome events or summary measures | Results – Tables 1–2 | pp. 11–13 |
| 16a | Give unadjusted estimates and precision (e.g., 95% CI) | N/A (exploratory) | – |
| 16b | Report category boundaries when continuous variables were categorised | Results – Table 1 | p. 12 |
| 16c | Consider translating estimates of relative risk into absolute risk | N/A | – |
